# Comparison of Inside-Out and Outside-In Methods of Femoral Tunnel Preparation in Anterior Cruciate Ligament (ACL) Reconstruction Using 3D-CT

**DOI:** 10.7759/cureus.23367

**Published:** 2022-03-21

**Authors:** Abhinay Vadlamudi, Amit Kale, Jaiman Sharma, Vishal Patil, Mukund Pai

**Affiliations:** 1 Orthopedic Surgery, Dr. D. Y. Patil Medical college, Hospital and Research Centre, Dr. D. Y. Patil Vidyapeeth, Pune, IND

**Keywords:** retrograde, transportal, 3d ct scan, knee surgery sports traumatology and arthroscopy, anterior cruciate ligament (acl) reconstruction

## Abstract

Introduction

Anterior cruciate ligament (ACL) reconstruction techniques continue to evolve and the need to address the more anatomical femoral tunnel placement of the graft is critical, and in our study, we assessed the placement of femoral tunnel via transportal and retrograde drilling techniques.

Material and methods

Sixty patients where n=31 for retrograde, n=29 for transportal were assessed via CT knee for the femoral tunnel aperture on the intercondylar ridge via high low and deep shallow direction ratio and interpreted accordingly.

Results

In our study, the femoral tunnel done via transportal method (n=29) has a deep shallow ratio range of 22%-47% and mean of 31.9±6.5, and graft is anatomical in 79%. The femoral tunnel done via the retrograde method (n=31) has a deep shallow depth ratio range of 11%-41% with a mean of 27.5±6.5 and graft is anatomical in 77% of the study group and the p-value means the ratio is 0.01 (significant). The femoral tunnel done via transportal method (n=29) has a high low ratio range of 19%-45% and mean of 32.9±6.3 and graft is anatomical in 72%. The femoral tunnel done via the retrograde method (n=31) has a deep shallow depth ratio range of 20%-38% with a mean of 33.9±4.1 and graft is anatomical in 94% of the study group with a p-value mean ratio being 0.51 (insignificant).

Conclusion

Watch out for the femoral tunnel placement in a deep shallow direction while going for standard transportal technique and high low direction while performing retrograde technique.

## Introduction

Anterior cruciate ligament (ACL) reconstruction techniques are evolving, and for the surgeon, technical considerations include graft selection, correct graft tensioning (as too taut will limit the range of motion, especially extension, and too loose will cause residual laxity), and correct tunnel placement, as it must provide sufficient stability during knee function without causing graft impingement within the intercondylar notch [1-3]. The surgeon's goal is to reconstruct the ACL as close to its natural state as possible. Surgical techniques have evolved over time, and the surgeon's current goal is “anatomic” reconstruction [4,5].

A surgeon will test whether or not the graft placement and tensioning were adequate, and the patient will be able to observe this when their activity levels increase. Thus, imaging is used in the early postoperative period to rate surgical success as “good” or “poor” depending on the location of the graft tunnels [6,7].

Many previous studies compared the femoral tunnel placement via transtibial, transportal, and outside-in methods, and many of them have given their own advantages and drawbacks of each technique, as well as radiographic assessment, and transtibial resulted in the more vertical and non-anatomical placement of the graft, while other two possess more or less anatomical placement of the graft when compared to past studies that have done to locate the anatomical landmarks of the ACL footprint [8-12].

Given the limitations of the preceding studies, the current study compares the femoral tunnel placed via standard transportal technique (inside out) and retrograde drilling technique (outside in).

## Materials and methods

The Institutional Ethics Sub-Committee (IESC) of Dr. D. Y. Patil Medical College and Research Centre in Pune approved this prospective study (research approval number: IESC/PGS/2019/93). We conducted this study on 60 patients admitted to the Department of Orthopedics at DPU, Pune, who were diagnosed with primary complete ACL rupture. All 60 patients provided informed written consent. This research was carried out between June 2019 and July 2021. Following pre-anesthesia check-ups, patients who were all medically fit for surgery were scheduled for arthroscopic reconstructive surgery of the ACL rupture.

Inclusion criteria

Patients who have been diagnosed with a primary complete ACL tear and are more than 18 years of age but less than 60 years of age were included in this study.

Exclusion criteria 

Patients who were medically unfit for surgery, patients who had failed primary ACL reconstruction surgery, patients who had multi-ligament injuries to the knee joint in addition to the ACL tear.

Selection criteria

All of the surgeries were performed by a single surgeon, with others assisting him at various times, and the randomization of the technique used was determined by paper chits totaling 60, with 30 of each technique written on them, which were chosen following the patient's admission and performed accordingly.

Surgical technique

Transportal (Inside-Out)

The ACL impression is depicted on the average surface of the lateral femoral condyle at 90 degrees of knee flexion, and the section point is identified. The entry area is then bored with an aide wire in 120 degrees of knee flexion using a femoral offset aimer inserted through the anteromedial port. Penetrating continues until the aide wire tip appears on the parallel side of the distal thigh near the femur epicondyle. The femoral passage was then created by drilling through both all over cortices with a 4.5 mm endoscopic drill bit. The length of the passage was then determined using a depth gauze. After that, the femoral passage was reamed with a reamer that corresponded to the graft diameter. Reaming was avoided 10-15 mm from the far cortex depending on the length of the graft.

Retrograde Technique (Outside-In)

The ACL impression can be seen on the average surface of the lateral femoral condyle at 90 degrees of knee flexion. The acufex pinpoint femoral aimer is placed at the near cortex of the lateral femoral condyle, 60 degrees to the sagittal plane and 20 degrees to the coronal plane, over the previous ACL impression. The femoral passage is bored with the acufex trunav until the previous leg tendon impression is reached, at which point the passage length is estimated using a burrow profundity assessment check. After estimating the length with a button flip scope of 5-10mm, retrograde boring is performed with a boring apparatus identical to the graft distance across, ending within 10mm of the far cortex.

Tunnel assessment

A 3D-CT scan of their knee will be performed in our institute within three days of surgery using a SIEMENS 128 slice (120Kv, 180mA) and 3D reconstructed image slices are isolated. The quadrant approach of Bernard et al [13] was used to determine the femoral tunnel position. The tunnels were measured from the deepest subchondral contour to the center of the tunnel and represented as a percentage of the intercondylar notch ceiling. A rectangular measurement frame is formed by the Blumensaat's line, a parallel line tangent to the most inferior margin of the femoral condyle, and two perpendicular lines tangent to the femoral condyle's deepest/subchondral contour.

**Figure 1:**
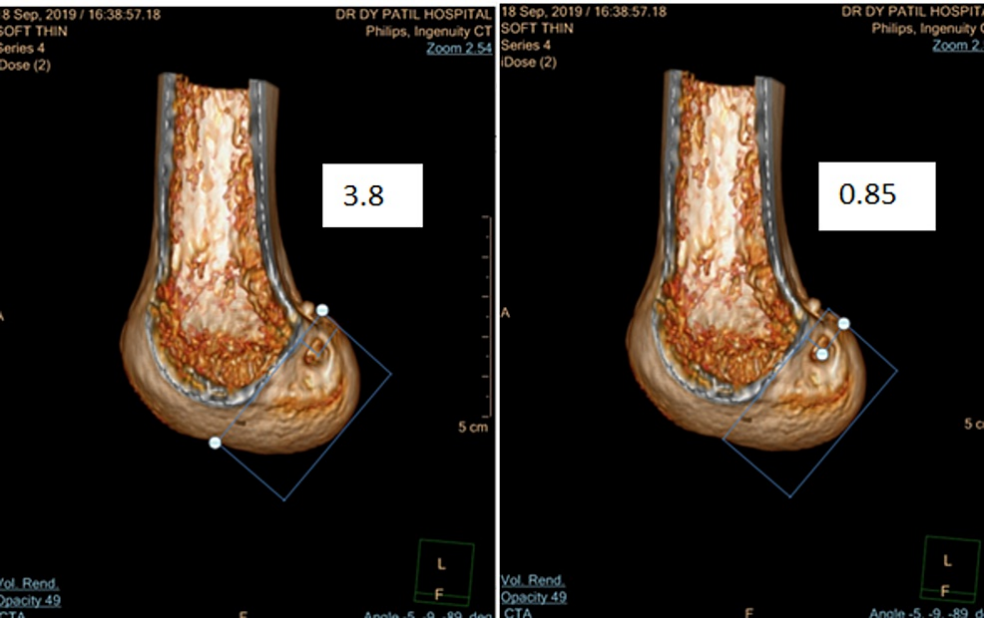
CT image of distal femur showing the tunnel assessment in deep-shallow position (left and right)

Figure 1 depicts the sagittal cut of distal femur and shows tunnel assessment in deep-shallow direction and the values in the white box represents the measurement of femur as a whole in first part, whereas the length of tunnel from deep in deep-shallow direction and the ratio of the tunnel length to total length is taken as previously explained by Bernard et al. [13].

**Figure 2:**
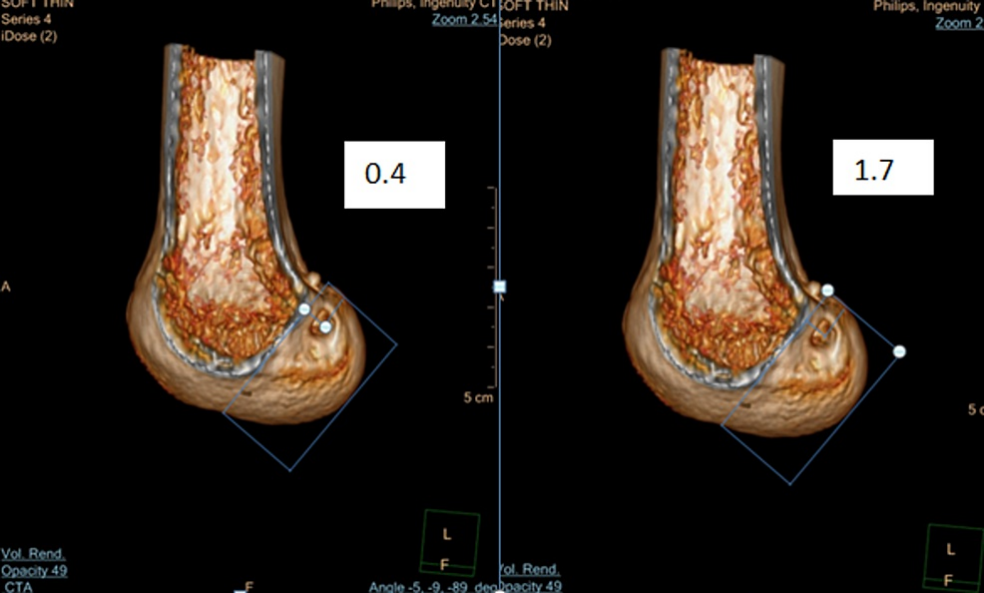
CT image of distal femur showing tunnel assessment in high-low position (left and right)

Figure 2 depicts the sagittal cut of distal femur and shows tunnel assessment in high-low direction and the values in the white box represents the measurement of tunnel from high in high-low direction in first part, whereas the whole length of tunnel in high-low direction in second part and the ratio of the tunnel length to total length is taken as previously explained by Bernard et al. [13].

## Results

**Figure 3:**
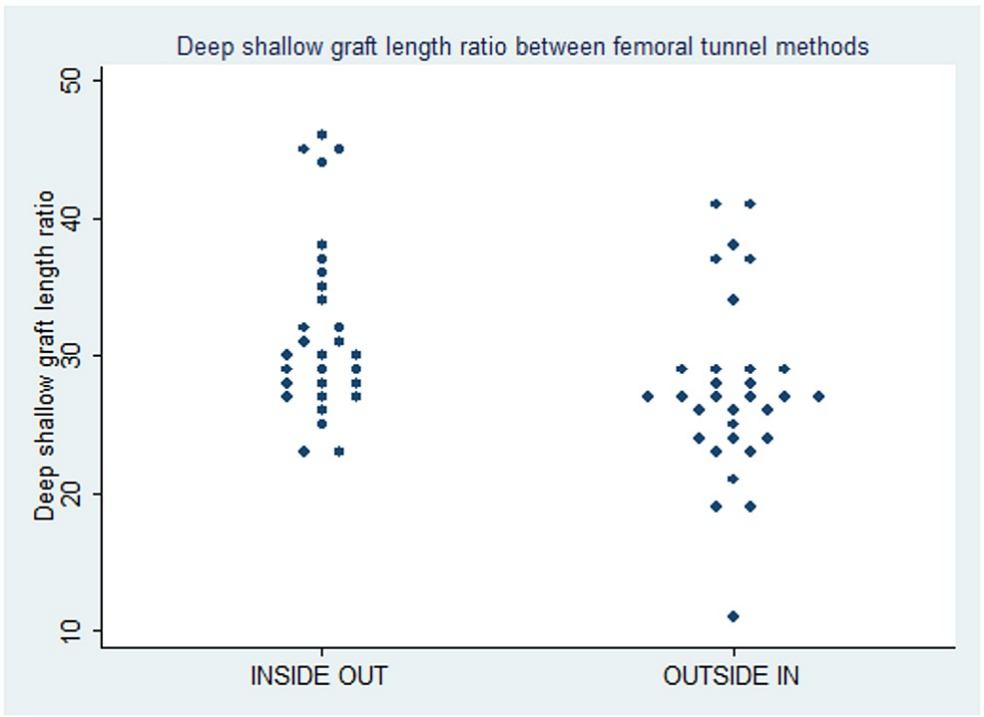
Scatter plot of subjects in the deep-shallow direction

Figure 3 shows a scatter plot of subjects in the study with normal values being 24%-37% [14]. Here the vertical axis represents the deep shallow graft ratio whereas the horizontal axis represents the various techniques of femoral tunnel placement and the dots represent various subjects.

**Figure 4:**
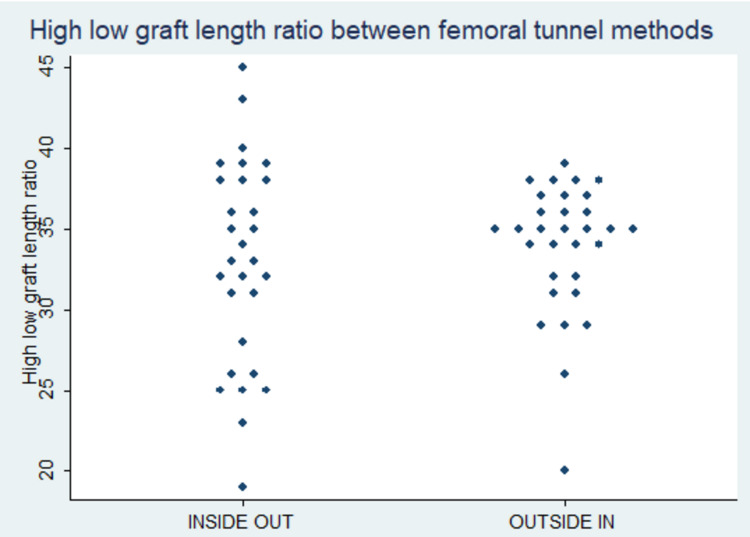
Scatter plot of subjects in high-low direction

Figure 4 shows a scatter plot of subjects in the study with normal values being 28%-43% [14]. Here, the vertical axis represents the high-low graft ratio whereas the horizontal axis represents the various techniques of femoral tunnel placement and the dots represent various subjects.

Table 1 shows the mean data of high-low graft length ratio of the population in the study with inside-out technique (n=29) being 32.9 (6.3) and outside-in technique (n=31) being 33.9 (4.1) with p-value being 0.51 and also demonstrates the comparison of deep shallow ratio between the two methods of femoral tunnel in which mean of inside-out technique is 31.9 (6.5) and outside-in technique is 27.5 (6.5) with p-value being 0.01 which is significant.

**Table 1:** Mean values and anatomical placement comparison *p-value < 0.05 is significant

	Transportal (inside out)		Retrograde (outside in)			P-value
	N=29	%	N=31		%	
Femoral (High-low)						
Mean	32.9 ± 6.3			33.9 ± 4.1		0.51
Anatomic	21	72	29		94	0.03*
Non-anatomic	8	28	2		6	
Femoral (deep-shallow)						
							Mean	31.9 ± 6.5			27.5 ± 6.5		0.01*
Anatomic	23	79	24		77	0.86
Non-anatomic	6	21	7		23	
Combined femoral grid						
Anatomic	16	55	22		71	0.22
Non-anatomic	13	45	9		29	

## Discussion

The study aims to measure the femoral footprints of ACL reconstruction using the inside-out and outside-in techniques. The study's main finding is that the majority of the measurements are accurate and correspond to normal anatomical footprints of the ACL.

The mean age in our study group was 29.7 (9.2) years, with a minimum of 17 and a maximum of 60 years. The interquartile range was 29-35.5 years, and the median age was 29 years. Many patients are young, with approximately 90% of the study group being under the age of 40, with elderly patients mostly presenting with mucoid degeneration of the ACL. In our study group of 60 patients, we had 73% males and 27% females, with a male to female ratio of 2.7:1. The group was nearly evenly divided on the side of the tear, with 52% on the right side and 48% on the left. Sports were the mode of injury in approximately 38% of cases, with trauma accounting for the remaining 62%.

The population in the study's mean ratio of high-low graft length ratio with inside-out technique (n=29) was 32.9 (6.3) and outside-in technique (n=31) was 33.9 (4.1). In our study, a femoral tunnel drilled from the inside out (n=29) had a deep shallow ratio range of 22%-47% and a mean of 31.9 (6.5), and the graft was anatomical in 79% of the cases. In contrast, a femoral tunnel done via outside-in drilling method (n=31) has a deep shallow depth ratio range of 11%-41% with a mean of 27.5 (6.5), and the graft is anatomical in 77% of the study group, with a p-value mean ratio of 0.01 (significant). Whereas the femoral tunnel done via inside-out drilling method (n=29) has a high low ratio range of 19%-45% and a mean of 32.9 (6.3), and the graft is anatomical in 72%, the femoral tunnel done via outside-in drilling method (n=31) has a high low depth ratio range of 20%-38% and a mean of 33.9 (4.1), and the graft is anatomical in 94% of the study group with the insignificant p-value.

Also, in our study, we found that the outside-in drilling technique has better graft placement in the high-low direction, with 94% being anatomical compared to 72% being anatomical in the inside-out technique with a p-value of 0.03 (significant) as compared to deep-shallow where the graft is anatomical in 79% of the inside-out group and 77% in the outside-in group with a p-value of 0.86 (insignificant).

The mean value is statistically significant in the deep-shallow position for the inside-out group, indicating that the graft placement has a higher chance of being anatomically placed, whereas the individual ratios and anatomically placed grafts in both study groups are statistically insignificant. Whereas the mean value of the graft in the high-low position is insignificant, the graft is more anatomically placed in the outside-in group with a significant p-value. The aimer pivot, where the guidewire may exit slightly off-center, is responsible for the above disparity in the deep shallow direction of the femoral tunnel placement.

Parkar et al. discovered that when comparing transtibial and anteromedial techniques, the p-values for the overall femoral grid and tibial tunnel, as well as isolated femoral high-low position, are significant and that the high-low direction is an important factor [14].

Nakamura et al. [15] compared post-operative CT of transtibial, transportal, and outside-in groups and concluded that the transtibial technique has non-anatomic tunnel placement compared to outside-in and transportal techniques, as well as an insignificant difference in portal placement between outside-in and inside-out techniques.

This study focuses on current novel techniques and their lack of agreement on which technique of femoral tunnel placement is better, and in our study we aimed at the anatomical placement of femoral tunnel.

Additionally, many studies compared the transtibial and anteromedial techniques of femoral tunnel placement and used different methods of assessment, which makes the conclusion and inference different based on the method [16,17].

Inter-rater agreement was not considered when evaluating the CT scan because previous studies on the subject demonstrated insignificant inter-rater and intra-rater variability [18,19].

Also, the evaluation of actual tunnel positioning was done after the surgery, and tunnel lengthening and widening were not considered. The sample size was smaller, in a single center, and performed by a single surgeon, and larger groups with multi-center studies are needed to assess the femoral tunnel placement techniques, as both mean values appear to be near normal, except for a few extremes in the outside-in drilling technique, which can be attributed to the newer technique being used by the current surgeon, which is a known complication of ACL reconstruction for surgeons in their early stages of learning [20].

## Conclusions

Although equally placed anatomically in both techniques, the femoral tunnel position by outside-in drilling technique is more anatomical in our study group in the high-low direction and has a significant mean ratio in the deep-shallow direction (more anatomical in inside-out drilling technique). In a combined femoral grid assessment of both techniques, the outside-in technique is more anatomically placed, despite the extremities of the outside-in technique in the deep-shallow direction, where the extremities can be attributed to the surgeon's newer method. So, both techniques have an equal chance of being anatomically placed, and it is up to the surgeon to decide. While using the inside-out technique, keep an eye on the high-low direction and consider the deep-shallow direction when using the outside-in technique.
